# The pivotal application of patient-derived organoid biobanks for personalized treatment of gastrointestinal cancers

**DOI:** 10.1186/s40364-022-00421-0

**Published:** 2022-10-08

**Authors:** Ya-ya Yu, Yan-juan Zhu, Zhen-zhen Xiao, Ya-dong Chen, Xue-song Chang, Yi-hong Liu, Qing Tang, Hai-bo Zhang

**Affiliations:** 1 Department of Oncology, The Second Affiliated Hospital of Guangzhou University of Chinese Medicine, Guangzhou, China; 2 Department of Oncology, Guangdong Provincial Hospital of Chinese Medicine, Guangzhou, Guangdong China; 3 Clinical and Basic Research Team of TCM Prevention and Treatment of NSCLC, Guangdong Provincial Hospital of Chinese Medicine, Guangzhou, China; 4 State Key Laboratory of Dampness Syndrome of Chinese Medicine, The Second Affiliated Hospital of Guangzhou University of Chinese Medicine, Guangzhou, China

**Keywords:** Gastrointestinal cancers, Patient-derived tumor organoids, Biobanking, Personalized anti-cancer therapy

## Abstract

Gastrointestinal cancers (GICs) occupy more than 30% of the cancer-related incidence and mortality around the world. Despite advances in the treatment strategies, the long-term overall survival has not been improved for patients with GICs. Recently, the novel patient-derived organoid (PDO) culture technology has become a powerful tool for GICs in a manner that recapitulates the morphology, pathology, genetic, phenotypic, and behavior traits of the original tumors. Excitingly, a number of evidences suggest that the versatile technology has great potential for personalized treatment, suppling the clinical application of molecularly guided personalized treatment. In the paper, we summarize the literature on the topics of establishing organoid biobanks of PDOs, and their application in the personalized treatment allowing for radiotherapy, chemotherapy, targeted therapy, and immunotherapy selection for GICs. Despite the limitations of current organoid models, high-throughput drug screening of GIC PDO combined with next-generation sequencing technology represents a novel and pivotal preclinical model for precision medicine of tumors and has a great value in promoting the transformation from basic cancer research to clinical application.

## Background

Cancer, the major health concern worldwide, caused almost 10.0 million deaths and 19.3 million new cases globally in 2020 [1]. Gastrointestinal cancers (GICs), which includes esophageal cancer (EC), gastric cancer (GC), colorectal cancer (CRC), primary liver cancer (PLC), biliary cancer (BC), and pancreatic cancer (PC), contribute to nearly one-third of the whole cancer-related death around the world [1]. Although improved treatment methods or strategies have lengthened the disease-free survival (DFS) of patients with advanced GICs to more than 2 years, the current cancer treatment strategies have had limited improvement in the overall survival (OS) [1, 2]. In conventional approaches, patients with the same tumor type receive the same treatment, which can be described as ‘one-size-fits-all’ treatments. However, management of advanced cancers using such a treatment strategy proves to be challenging, with marked heterogeneous therapeutic responses to radiotherapy, chemotherapy, targeted therapy, immunotherapy, and, a combination of them across individual patients, which finally restricts the improvement of OS [3]. Currently, personalized medicine, meaning ‘one drug and one dose, one patient’ treatment, is progressively improving the tumor patient outcomes for its better characterization of the pharmacogenomic and molecular traits of tumors. Genomics promote personalized medicine by providing the mutational change information of tumor tissues but fail to precisely predict whether the patients will benefit from the specific genomics-based treatments in clinic. Therefore, a preclinical model, that can carry the genetic information of the primary tumor and provide an assessment of drug response to anti-cancer therapy is urgently required for precision treatment.

Both patient-derived tumor xenografts (PDTXs) and cancer cell lines have long been the classic preclinical models for anti-cancer drug research. However, many drawbacks hamper the models for precision treatment. The immortalized cancer cell lines have huge disadvantages of unable to retain vital features and keep the genetic heterogeneity of original tumors [4–6]. While the low success rate and high cost PDTX model has the drawbacks of experiencing mouse-specific tumor evolution, and failing to perform high-throughput drug screening at a clinically meaningful time window [7, 8]. Therefore, there is an urgent need to unlock new high-efficiency preclinical models that can accurately replicate tumor patient information. Recently, a three-dimensional (3D) organoid model, which can cover the demands of the high-efficient established rate and reserve the original tissue features, has been successfully developed. Patient-derived organoids (PDOs) of tumors, which can reserve the features of original tumors from patients, have great value in improving basic and clinical cancer research, especially in personalized treatment [9, 10]. Inspiringly, the protocols of PDO models have been established from different organs and various cancer types, such as the brain [11], lung [12], prostate [13] and the breast [14]. Among them, the study of PDOs from GICs, including GC, CRC, PLC, PC, BC and, EC is involved in the most mature research [15–17].

**Fig. 1:**
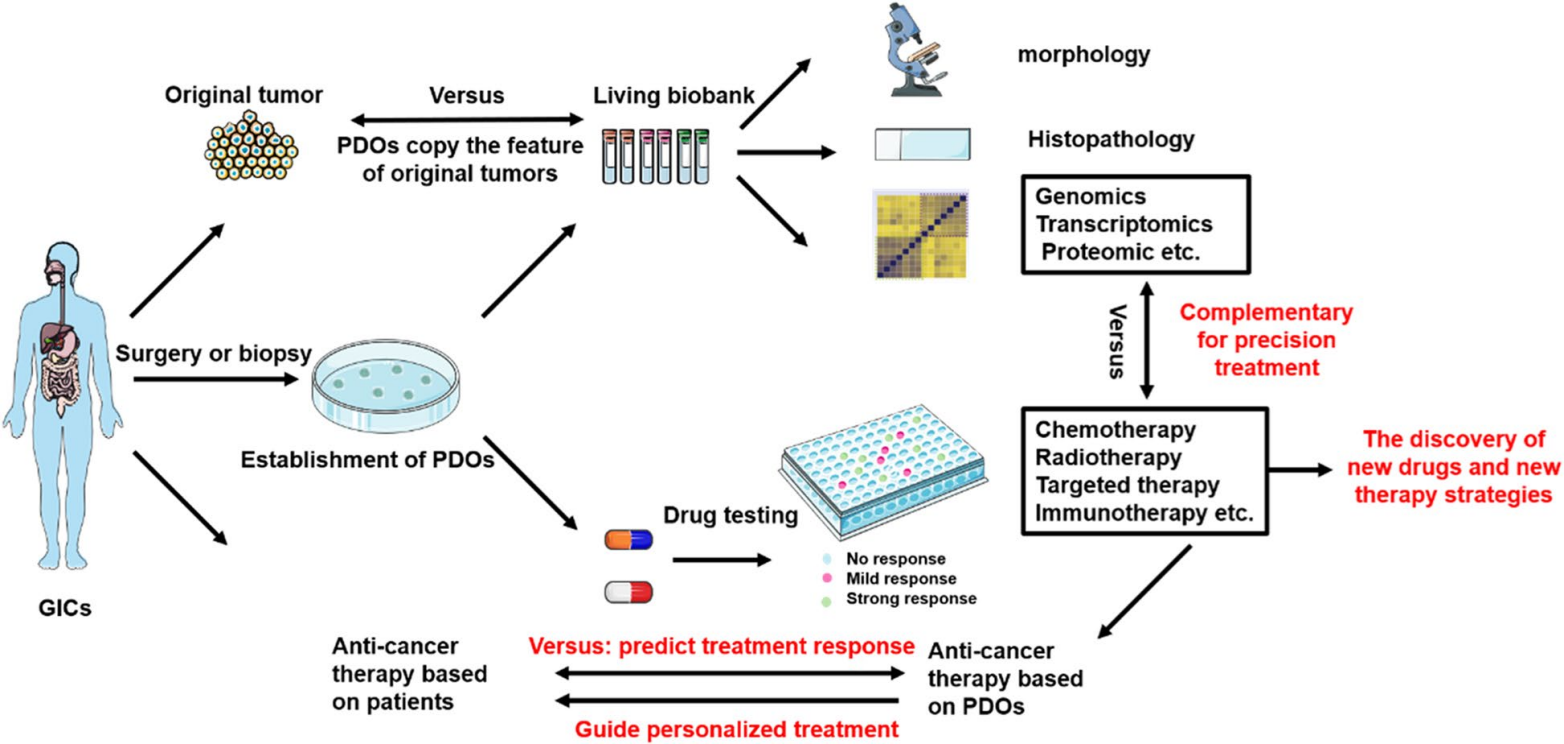
The flow chart of the establishment of living biobanks of GIC PDOs and the application of GIC PDOs in personalized treatment. Notes: PDOs of GICs can be cryopreserved and stored in the living organoid biobanks for cancer research. PDOs of GICs recapitulate their morphology, pathology, and, genetic traits of the original tumors (upper panel). The PDO technology has the application of personalized treatment allowing for chemotherapy, radiotherapy, targeted therapy, immunotherapy, or the combination of their selections for an individual patient with GICs (lower panel). PDOs-Patient-derived organoids. GIC-Gastrointestinal cancer

In this review, we describe current biobanks established from GIC PDOs and PDO- xenografts (PDO-Xs, the in vivo model of PDOs), and underline the potential applications of PDOs for personalized treatment. The flow chart of the establishment of living biobank and the application of PDOs for personalized treatment of GICs is shown in Fig. 1.

## The successful establishment of living biobanks of GIC PDOs

Organoids, upon embedment into 3D matrices and grown into self-organizing organotypic structures from tissue-derived adult stem cells (ASCs) with relative high efficiency, led to the breakthrough in novel cancer models. Sato et al. firstly reported that a leucine-rich repeat-containing G protein-coupled receptor 5 positive LGR5 (+) mouse crypt stem cell could successfully generate intestinal organoids in 2009 [18]. Since then, organoid technology, bridging the 2D in vitro models and in vivo models, has sprung up and has shown potential for oncology research [19]. PDOs from human tumor tissues enable the establishment of tumor ‘living biobanks’, in which organoid cultures represent disease diversity in pathological subtypes and genotypes [20–23]. The successful establishment of living biobanks contains the long-term culture, passage and cryopreservation of PDOs from tumor tissues and the consistency of morphology, histology, pathology, genetic, phenotypic, and behavioral traits between organoids and their original tumors. For GICs, the living biobanks have been established from CRC, PLC, PC, EC, BC and GC within various histological subtypes and genotypes [15–17].

The ability to generate organoids from healthy mouse and human ASCs has paved the way to grow PDOs from tumor tissues. The combination of R-spondin 1 (a ligand of LGR5 and Wnt agonist), Noggin (a bone morphogenetic protein inhibitor), and EGF could mimic the in vivo stem cell niche in a serum-free 3D matrix, thus supporting the proliferation and differentiation of LGR5 (+) mouse intestinal stem cells as 3D epithelial structures [18]. Additional components were required for human gut organoids: Wnt, SB202190 (a p38 inhibitor) and A83–01 (a TGF-β inhibitor). This organoid culture composition also supports the expansion of CRC PDOs and some modifications allowed the successful establishment of PDOs of other GICs [15, 24]. Stem cell niche components, small molecule inhibitors, and growth factors in PDOs of GICs culture medium majorly include Wnt-3A, Noggin, R-spondin-1, B27, N2, nicotinamide, N-acetylcysteine (NAC); Y27632 (the RHO kinase inhibitor), A83–01 and SB202190; EGF, FGF10, FGF7, hepatocyte growth factor (HGF), gastrin, prostaglandin E2 (PGE2). There are some differences in organoid culture medium supplements among different cancer types and different pathological types of GIC PDOs mainly based on the specific genetic background and specific growth conditions [20, 25]. The sample sources, number of cases, niche factor supplements (except for NAC, B27, N2, and nicotinamide), success establish rates, and key findings of living biobanks from each literature on GICs are summarized in Table 1.

**Table 1:** The establishment of GIC PDO living biobanks

Cancer type	Sample source	No.	Niche factor supplements	Success rate	Key findings	Ref
CRC	Surgery	20	Wnt, Noggin, R-Spondin, EGF, Gastrin, A83–01, SB202190, PGE2	81%	The features of genetic changes in CRC PDOs largely resembles the mutational analyses of CRC tissues.	[22]
CRC	NM	55	Wnt-3A, Noggin, R-spondin-1, EGF, Gastrin I, A83–01, SB202190	100%	CRC PDOs of various pathological types have been established. The histopathological grading and differentiation of CRC PDOs were nearly the same as those of their parental tumors in vitro and in vivo.	[23]
CRC	Biopsy and surgery	35	Wnt-3A, Noggin, R-spondin-1, EGF, Gastrin, A83–01, SB202190	60%	PDXs appear closer to the CRC molecular groups than PDOs. PDOs has less complex molecular subpopulations than PDXs due to their loss of matrix and higher expression of xenobiotic and fatty acid processes related genes.	[26]
CRC	NM	7	Wnt, R-Spondin, Noggin, EGF, Gastrin, A83–01, SB202190, PGE2	NM	The individualized patient-specific genomic and proteomic profiles of CRC PDOs may help the disease diagnosis	[27]
CRC	NM	91	Wnt-3A, Noggin, R-spondin-1, EGF, Gastrin, A83–01, SB202190	NM	The therapeutic responses and inhibitor effects on the oncogene related signal pathways to the CRC PDOs were widely variable.	[28]
mCRC	Biopsy	14	Noggin, R-Spondin, EGF, Gastrin, A83–01, SB202190, PGE2	71%	Nearly 90% of somatic mutations are shared between PDOs and matched tumors. None of the mutations that were found in either CRC tissues or CRC PDOs were genes amenable for drug targeting or in tumor driver genes.	[29]
mCRC	Surgery	3	Noggin, EGF, Gastrin, A83–01, SB202190	NM	To test the efficacy of PARP inhibitors in mCRC patients who carry HR deficient tumors and have experienced tumor shrinkage upon induction of FOLFOX-chemotherapy.	[30]
mCRC	Biopsy	40	Wnt-3A, Noggin, R-spondin-1, EGF, Gastrin, A83–01, SB202190	63%	CRC PDOs can be applied to predict the drug response of corresponding CRC patients to CPT-11-based chemotherapy.	[31]
RC	NM	65	Wnt-3A, R-spondin-1, EGF, Gastrin I, A83–01, SB202190.	77%	RC PDOs retain molecular features of the original tumors.	[32]
RC	Biopsy	96	Noggin, R-spondin 1, EGF, Gastrin, A83–01, SB202190, PGE2	86%	A living biobank was generated from advanced RC patients treated with neoadjuvant chemoradiotherapy in a phase III clinical trial.	[33]
PC	Biopsy and surgery	8	Wnt-3A, Noggin, R-spondin1, EGF, Gastrin, FGF10	80%	The tumor development progress from early-grade tumor formation to locally invasive carcinomas and even metastatic carcinomas is reappeared in the PC PDO-X model.	[20]
PC	Biopsy and surgery	101	Wnt-3A, Noggin, R-spondin-1, EGF, Gastrin I, FGF10, PGE2	73%	The gene mutational spectrum and transcriptional subtypes of PC PDOs are largely the same as those of human PC tissues. Novel driver oncogenes and unique clusters are identified based on PC PDOs.	[34]
PC	Biopsy and surgery	52	Noggin, R-spondin, EGF, Gastrin, A83–01, PGE2, FGF10	63%	The PC PDOs copy the histology and typical genetic alterations of human PC tissues. Drug screening of 76 new drugs provides evidence for the drug’s effectiveness in the clinic.	[35]
PC	Surgery and biopsy	44	Wnt-3A, Noggin, R-spondin1, EGF, Gastrin, FGF10	NM	The PDO-based prediction model successfully predicts the response in treatment-naive patients for front-line regimens but fails to predict the response in pretreated patients.	[36]
PC	Biopsy	10	Wnt-3A, Noggin, R-spondin1, EGF, Gastrin, A83–01, Y-27632, FGF10	NM	The mutational spectrum in PC PDO supernatants recapitulates this in the human PC tissues, which facilitates drug screening of PC PDOs in a shortened time frame.	[37]
PDAC	Surgery	17	Y-27632, FGF2, hydrocortisone, all-trans retinoic acid, Ascorbic acid, Insulin	85%	PDAC PDOs recapitulate the differentiation status, histology, phenotypic heterogeneity patient-specific physiologic changes of parental tumors.	[38]
PDAC	Biopsy and surgery	39	Wnt-3A, Noggin, Rspondin-1, Gastrin, FGF10	80%	Three functional subtypes based on the dependencies on R-spondin and Wnt are confirmed. The heterogeneity of Wnt niche independency of PDAC forms in tumor progression.	[21]
PDAC	Biopsy	25	Wnt-3A, Noggin, R-spondin1, EGF, Gastrin I, A83–01, PGE2, FGF10	67%	PDCA PDOs were successfully established using EUS-FNB at the time of initial diagnosis.	[39]
PDAC	Biopsy	18	Wnt-3A, Noggin, R-spondin1, EGF, Gastrin I, A83–01, Y-27632, FGF-10	83%	The drug screening of PDAC PDOs can inform therapeutic selection and patient stratification for PDAC patients, and identify gene signatures associated with new therapeutic response combined with omics data.	[40]
PDAC	Surgery	6	Wnt-3A, R-spondin1, EGF, Gastrin I, A83–01, Y-27632, FGF-10	NM	Nine metabolites in early recurrent PDAC PDOs are increased when compared with late recurrent PDOs, indicating that an increased anaplerotic metabolism and energy metabolism fasten the PDAC recurrence.	[41]
GC	Biopsy and surgery	15	Wnt-3A, Noggin, R-spondin-1, EGF, Gastrin, FGF10	NM	The genomic profiling of paired human GC tissues and GC PDOs is largely the same, including the similar KRAS alterations.	[42]
GC	NM	37	Wnt-3A, Noggin, R-spondin1, EGF, A83–01, FGF10, Nutlin-3	NM	Generation and analysis of GC PDOs reveal molecular signatures underlying distinct histopathological subtypes and independence of Wnt signaling.	[43]
GC	NM	46	Wnt-3A, Noggin, R-spondin-1, EGF, FGF10, Gastrin, A83–01, Y-27632	> 50%	A biobank of GC PDOs with distinct subtypes is established and the PDOs maintain similarity to the parental tumors for long.	[44]
GC	Surgery	24	Wnt, Noggin, R-spondin-1, EGF, Gastrin, FGF10, A83–01, Y-27632	NM	The living bank of GC PDOs may predict therapy response for individual patients.	[45]
GC	Surgery	7	Wnt, Noggin, R-spondin-1, EGF, Gastrin, FGF10, Y-27632	NM	RNA sequencing reveals that the PDOs closely resemble the primary tumor tissue.	[46]
GC	MA	11	Wnt-3A, Noggin, R-spondin1, EGF, Gastrin, A83–01, Y-27632, FGF10	92%	GC MADOs copy the histology, and genomic feature of the original MA cancer cells.	[47]
PLC	Surgery	8	EGF, Gastrin I, A83–01, FGF10, HGF, FSK, Y-27632, dexamethasone	47%	PDOs of PLC (including HCC, CAC, and CHC) copy the histology and gene signature of the parental human PLC tissues.	[25]
PLC	Surgery and biopsy	27	Wnt-3A, Noggin, R-spondin, EGF, Gastrin, A83–01, FGF-10, HGF, FSK	NM	Drug screening of 129 drugs was performed using PLC PDO model.	[48]
HCC	Biopsy	10	Wnt-3A, Rspondin-1, Gastrin, EGF, A83–01 FGF10, HGF, FSK	26%	HCC PDOs maintain the morphology, HCC tumor markers and genetic heterogeneity of the original human HCC tissues.	[49]
EADC	Surgery	10	Wnt-3A, Noggin, R-Spondin-1, EGF, A83–01, SB202190, FGF10	31%	EADC PDOs maintain the morphology and molecular signature of the primary human EADC tissues. EADC PDOs and the original tumor tissues have the same clonal architecture.	[50]
ESCC	Biopsy	11	Wnt-3A, Noggin, R-Spondin, EGF, Gastrin, A83–01, SB202190	69%	ESCC PDOs recapitulate the histopathologic features of the original tumor tissues. Successful ESCC PDO generation is positively connected with poor response to radiation, chemotherapy and neoadjuvant chemotherapy.	[51]
BC	Surgery	6	R-spondin-1, EGF, Gastrin, A83–01, FSK, Y-27632	NM	The long-term cultured BC PDOs recapitulate the histopathology, gene signature of the original BC tissues.	[52]

*PDO* Patient-derived organoid, *GIC* Gastrointestinal cancer, *No.* Number of samples, *Ref* Reference, *CRC* Colorectal cancer, *PGE2* Prostaglandin E2, *NM* Not mentioned, *mCRC* Metastatic colorectal cancer, *RC* Rectal cancer, *GC* Gastric cancer, *EGD* Esophageal gastroduodenoscopy, *RC* Rectal cancer, *PC* Pancreatic cancer, *PDO-X* Patient-derived organoid- xenograft, *PDAC* Pancreatic adenocarcinoma, *MA* Malignant-ascites, *MADOs* Malignant-ascites derived organoids, *HCC* Hepatocellular carcinoma, *FSK* Forskolin, *PLC* Primary liver cancer, *CAC* Cholangiocarcinoma, *CHC* Combined HCC/CAC, *EADC* Esophageal adenocarcinoma, *ESCC* Esophageal squamous cell carcinoma, *HGF* Hepatocyte growth factor, *CPT-11* Irinotecan, *EUS-FNB* Ultrasound-guided fine-needle biopsy, *BC* Biliary cancer

### Colorectal cancer (CRC)

CRC ranks third in terms of cancer-related incidence and ranks second in terms of cancer-related death worldwide [1]. CRC PDOs have been successfully propagated from various histological subtypes and even rare histological subtypes, such as neuroendocrine carcinoma and mucinous adenocarcinoma, and can be generated from both tumor resection specimens and tumor tissue biopsy sample with high success rates, ranging from 60 to 100%, shown in Table 1 [23, 29]. Fujii et al. generated a living biobank consisting of 55 CRC PDOs with various histological subtypes and clinical stages, and they discovered that the proliferation of CRC PDOs was affected by Wnt3A, R-spondin-1, SB202190 and oxygen concentration [23]. Recently, a living biobank consisting of 20 genetically diverse normal tissue-derived organoids and their corresponding CRC PDOs was generated [22]. Multiple collections of living biobanks of CRC PDOs showed largely resemblance with the original tumors in terms of histology, differentiation, genomic signature, transcriptomic profiling and proteomics [22, 23, 53]. Schumacher et al. demonstrated that CRC PDOs of CRC kept the adenoma-like architecture and the typical expression of CRC markers [28]. The results showed that CRC PDOs and CRC tissues shared common driver mutations in TGF-β, PI3K/AKT and EGFR/RAS/RAF/MEK and signaling pathways [28]. Yao et al. reported that rectal cancer (RC) PDOs showed a large resemblance with the matched RC tissues in the aspects of histology and typical marker expression [33]. Moreover, RC PDOs recapitulated the copy number variation (CNV) pattern and DNA copy number losses/gains of paired RC tissues in cancer driver genes [33]. The RC PDOs retained the gene mutation spectrum observed in original tumors in the most frequently mutated genes in RC with a nearly 95% overlap [33]. Besides these living biobanks, metastases CRC (mCRC) PDOs have also been generated [29]. It was found that 90% somatic mutations were shared between the mCRC PDOs and original mCRC tissues, and the DNA copy number outlines of the PDOs and matched tissues showed a correlation of 0.89 [29]. More meaningful, none of the mutations that were found in either mCRC tissues or mCRC PDOs were genes amenable for drug targeting or in tumor driver genes [29]. Distinct proteomic signatures were detected between PDOs of CRC tissues and corresponding healthy tissues and among CRC PDOs from individual patient [27]. The data reveal that the individualized patient-specific genomic and proteomic profiles of CRC PDOs may do help in personalized medicine [27]. The comparison of the proteomic profiles between CRC PDO and the original CRC tissues to ensure the preservation of proteomic profiles should also be made in the future. PDO xenografts (PDOXs) can be used to confirm in vitro finding in vivo [23]. It is important to find that CRC PDOs can keep the features of the original CRC tissues both in vitro and in vivo using the PDOX model [23]. In a study, both PDOs and PDXs from patients with various cancers, including CRC, played conservation of genomic feature and histopathology of the original tumors, including the lumina formation [10]. However, Moritz Schutte et al. reported that CRC PDXs appeared closer to the human CRC molecular groups than CRC PDOs [26]. CRC PDOs had less complex molecular subpopulations than PDXs due to their loss of matrix and higher expression of xenobiotic and fatty acid processes related genes [26].

### Pancreatic cancer (PC)

The mortality rates of PC remain high these years [54]. Pancreatic ductal adenocarcinoma (PDAC), accounting for nearly 85–90% of PC, has only about 10% of 5-year survival [54]. PDOs of PDAC has been successfully established in multiple studies, with the success rate ranging from 63 to 85%, shown in Table 1 [20, 21, 55]. The successful rate of long-term maintenance of PDAC PDOs was even high up to 66% by means of biopsy [39]. Seino et al. established a genetically characterized living biobank consisting of PDAC PDOs from 39 patients [21]. They reported that EGF should be eliminated for the enrichment and maintenance and of PDAC PDOs with KRAS-mutant gene [21]. Three PDAC PDOs subtypes were confirmed based on the dependencies on R-spondin and Wnt, which were associated with different genotypic characteristics, suggesting that the genetic background of PDCA affect the compositions of tumor PDOs culture medium [21]. The PDCA PDOs that were sensitive to the elimination of EGF could select TP53-mutants organoids, and nutlin3 (an inhibitor of MDM2) or Noggin removal could be helpful to select SMAD4-mutant PDOs [21]. PC PDOs showed largely resemblance with the original human PC tissues in the terms of histology, genomic profiling and transcriptomic features in vitro and in vivo. The most common driver-gene alterations in human PC, including KRAS, TP53, CDKN2A and SMAD4 were detected in corresponding PC PDOs [21]. Tiriac et al. reported that PDOs recapitulated the mutational and transcriptional spectrum of the matched PC tissues [34]. Driehuis et al. observed that PC PDOs retained the histology and carried genetic alterations of original human PC tissues using histology, RNA sequencing, and DNA sequencing methods in vitro [35]. A living bank of PDAC PDOs from 17 patients maintained the histology of primary PDAC tissues both in vitro and in vivo [38]. The observation that PDAC PDOs formed tumors in vivo like the derived tumors confirmed by another team [21]. Besides these living biobanks, PDOs of primary pancreatic intraepithelial neoplasms from both resected tumors and biopsies were also generated [20]. Orthotopically transplanted PC PDOs recapitulated the tumor development progress through early-grade tumor formation to locally invasive carcinomas and even metastatic carcinomas formation [20]. A study highlights that both PDXs and PDOs models of PDAC can preserve the molecular features of human PDAC tissues, indicating that PDOs can be applied for selective analysis over distinct levels of genomic complexity [56]. The success rate of generating PDOX model from 35 metastatic PDAC patients was nearly 50% using biopsy samples [57]. The metastatic PDAC PDO samples showed the same ability to metastasize to distant organs as derived patients [57]. The organoids from tissues of PDAC PDO-Xs preserved the KRAS mutational condition and epithelial characteristics of the original human PDAC tissues [57].

### Gastric cancer (GC)

According to data published in 2021, the cancer-related incidence rate of GC ranks the fifth and the cancer-related mortality rate of GC ranks the fourth worldwide [1]. Organoid culture technology has also been applied in GC [42, 43, 45, 53]. GC PDOs with different histological, molecular and phenotypic patterns were established [43]. The study showed that different genetic and epigenetic pathways could develop R-spondin/WNT niche independency in the GC PDOs [43]. For example, the addition of ZNRF3 and RNF43 mutations into GC PDOs was able to grant the independency of R-spondin [43]. Interestingly, TP53 and CDH1 mutation enrichment was found in R-spondin-independent GC PDOs with intact ZNRF3 and RNF43 [43]. Another living biobank of GC PDOs was established from biopsies and surgical tissues from 5 patients [42]. The genomic profiling of human GC tissues and paired GC PDOs is largely the same, including the similar KRAS alterations [42]. Seidlitz et al. generated GC PDOs from four subtypes of GC and found that the GC PDOs copied most characteristics of the original tumors, such as structure, the expression of typical GC markers and the common mutations in GC (for example, PI3K, ERBB2 and TP53) [45]. GC PDOs could also be generated from malignant-ascites (MA), and MA-derived organoids (MADOs) kept the morphology, histology and genomic profiles of the original MA tumor cells [47]. GC PDOs could preserve the features of the primary human GC tissues not only in vitro but also in vivo. Transplantating GC PDOs into immuno-deficient mice allowed the tumor formation with similar features as the corresponding human GC tissues [46]. Yan et al. generated GC PDOs from 34 patients that comprised normal, dysplastic, tumor and lymph node metastases, and the histology and molecular features of the PDOs remained analogous to in vivo tumors [44].

### Primary liver cancer (PLC)

PLC, the sixth most common cancer, causes the third most common cancer-related death worldwide [1]. PLC can be classified as either hepatocellular carcinoma (HCC), cholangiocarcinoma (CCA), or HCC-CCA (CHC), and HCC accounts for up to 80% of all cases of PLC. PLC PDOs of different types from resection or biopsy specimens have been established [25, 49]. There were two types of medium for the PLC organoids culture: classical human liver organoid culture medium and tumoroid-specific culture medium [25]. One PLC organoid could only grow in classical human liver organoid culture medium for its need of R-spondin-1 [25]. The remove of Noggin, R-spondin-1, and Wnt-3A, and dexamethasone and Y-27632 addition, were done to suppresses the normal cell growth, and the Y-27632 was only added during the first about 2–3 weeks of culture [25]. PLC PDOs showed largely resemblance with the human original PLC tissues in terms of histology, mutational and transcriptomic spectrum. PLC organoids largely recapitulated their original tumors even after long-term expansion at a histological level [25]. Solid architectures and pseudoglandular rosettes, the histological characteristic of HCC, were observed in the HCC PDOs and CHC PDOs [25]. While glandular regions with tumor cells invaded the lumen and grew in a cribriform pattern were observed in both CCA PDOs and human CCA tissues [25]. Moreover, high expression of HCC markers was found in PDOs of HCC, while CCA organoids expressed enhanced CCA markers [25]. PLC organoids faithfully recapitulated the alterations of their corresponding original tissues at transcriptomic level [25]. Driehuis et al. reported that the PLC PDOs preserved the histology and genetic alterations of the original human PLC tissues [35].

### Esophageal cancer (EC)

EC, major divided into esophageal adenocarcinoma (EADC) and esophageal squamous cell carcinoma (ESCC), still has a high mortality worldwide [1]. The ESCC PDOs were successfully established from 15/21 patients and kept the histological features of the human primary ESCC tissues [51]. The ESCC PDOs were composed of atypical and highly proliferative cancer cells with high nuclear-to-cytoplasmic ratio [51]. Moreover, more than 40% of ESCC PDOs showed dysregulated accumulation of TP53, the key feature of human ESCC [51]. Some studies have turned to generated EADC organoids using tissues from Barrett’ esophagus (BE, the premalignant condition of EADC) [15, 58]. The establishment of long-term BE PDOs was achieved in the condition with 20% R-spondin-1 conditioned medium and 50% Wnt-3A conditioned medium, and the addition of PGE2 [58]. The fact that the morphology, genomic and transcriptomic features of EADC PDOs were largely the same as those of the original tumors was confirmed [50].

### Biliary cancer (BC)

BC is one of the most aggressive cancers and patients with BC have very poor prognosis [1]. PDOs of BC have been successfully established and can be long-term maintained from gallbladder cancer (GBC) and neuroendocrine carcinoma of the ampulla of Vater, and they preserve the histopathologic features, genomic profiling of in the huaman original tumor tissues [52]. Shiihara et al. successfully cultured 30 PDOs of pancreato-biliary cancers (PBC), and found that most of the PBC PDOs showed identical genomic aberrations as those of the primary tumors [59].

Overall, the establishment of living biobanks of GIC PDOs can be a precious bioresource for basic and clinical research for the remarkable advantages of GIC PDOs, including keeping the properties of the human original tumors and high proliferative ability in vitro*,* especial for CRC and PC. PDOs of CRC, PC, GC, PLC and EC can be successfully established not only from surgical samples but also from biopsy specimens. Wnt-3A, R-spondin-1, Noggin, EGF, Gastrin, A83–01, and SB202190 are the most frequently used niche factor supplements for the culture of PDOs of CRC. Besides these niche factor supplements used in CRC, additional use of FGF10 and Y-27632 for the culture of PDOs of GC, FGF10 and PGE2 for PC, FGF10, HGF and FSK for PLC, have been reported. More importantly, most studies have shown that GIC PDOs both genetically and phenotypically resemble the human original tumor tissues in vitro and in vivo, making the GIC PDO biobanking an important preclinical model for personalized medicine [22, 60, 61].

## GIC PDOs as an advantageous preclinical model for personalized treatment

Personalized medicine is progressively improving the prognosis of cancer patients for its better characterizating the pharmacogenomic and molecular features of tumor tissues. The technologies of Gene sequencing and PDO-based drug susceptibility testing promote the development of precision medicine. As shown above, PDOs of GICs are relatively easy to be established and can preserve characteristics in physiology, pathology, phenotype, genotype, and transcriptome of the human original tumor tissues [20, 29, 39, 42, 49]. Moreover, PDOs generated from different sites of the same patient can better simulate intra-tumor heterogeneity, making it possible and reliable to improve the anti-cancer therapy for individual patients [62, 63]. Large numbers of evidence have provided a proof of concept for applying GIC PDO model to personalized therapy of cancer [22, 23, 34]. Overall, the high-throughput drug screening of GIC 3D-PDO model has the potential to fill the gap between human GIC cell lines and clinical trials. The application PDOs of GICs in the personalized treatment allows for the radiotherapy, chemotherapy, targeted therapy, and immunotherapy selection for GIC patients.

## GIC PDOs as an advantageous preclinical model for radiotherapy and chemotherapy

The choice for radiotherapy and chemotherapy is mainly based on the patient’s cancer types, histological types, and stages, and sometimes even based on the investigator preference. Currently, for traditional treatments such as chemoradiotherapy, patients with specific tumor types are generally treated with the mimic regimen, namely the ‘one-size-fits-all’ treatments. However, cancer patients exhibit distinct responses to chemotherapeutics and radiotherapy in the real clinical world. The choice of the best treatment strategies using radiotherapy and chemotherapy, and the choice of effective treatment strategies after drug-resistance, are largely lacking basis. Methods that can accurately predict the effect of radiotherapy and chemotherapy are urgently needed. Chemoradiotherapy screening of GIC PDOs can help to choose more suitable treatment methods for individual patients. There have been prospective studies and cohort studies showing that screening of radiotherapy and chemotherapy based on PDOs of GICs can well predict the clinical efficacy of patients. Furthermore, PDOs of GICs can help to find new treatment strategies for GIC patients with clinical radio-chemotherapy resistance. Overall, GIC PDO models have great potentials for the precision treatments in choosing radiotherapy and chemotherapy, which are summarized in Table 2.

**Table 2:** Precision treatment for chemoradiotherapy using GIC PDOs

Cancer type	Chemotherapy drugs	Assay	Key findings	Ref.
CRC	OXA, 5-FU, DDP, CPT-11, DOC, GEM	CTG	Organoid technology allows personalized treatment design for chemotherapy.	[22]
CRC	5-FU, OXA	CTG	Drug response between PDOs and PDXs were fairly concordant for OXA but were inconsistent for 5-FU.	[26]
CRC	5-FU, OXA, CPT-11, Capecitabine, Folinic acid	CTG	The sensitivity, specificity, and accuracy rates of the CRC PDOs for predicting chemotherapy responses are 63.33, 94.12, and 79.69%, respectively.	[64]
CRC	OXA	Single-Cell RNA-Seq	The technologies of Single-cell RNA-Seq and drug-screening based on CRC PDOs help to find cancer heterogeneity.	[65]
CRC	Raltitrexed, OXA, MMC, GEM, 5-FU, Lobaplatin, Abraxane	CCK-8	Raltitrexed has the most significant hyperthermia synergism among the common hyperthermic intraperitoneal chemotherapy drugs in CRC PDOs.	[66]
mCRC	5-FU, OXA, CPT-11	CTG	The drug tests based on mCRC PDOs successfully predict the drug response to CPT-11 but fail to predict drug response to 5-FU plus OXA.	[31]
mCRC	5-FU, OXA, CPT-11, SN-38	CTG	mCRC PDOs show sensitivities to 5-FU, SN-38, the same as drug responses in clinic.	[67]
mCRC	Radiation, 5-FU, OXA	Optical metabolic imaging	The drug screening of mCRC PDOs shows promise to predict chemotherapy/radiation sensitivity for patients. It prospectively predicts response for a mCRC patient treated with re-treatment of FOLFOX chemotherapy.	[68]
mCRC	MMC, OXA	Live-cell imaging	Peritoneal metastasis-derived organoids can be applied to evaluate HIPEC regimens for mCRC patients.	[69]
mCRC	5-FU, OXA, CPT-11	CTG	The mCRC PDO-^Sponge^ model keeping the similar expression level of lamin-A as their primary tumor tissues successfully predict FO chemotherapeutic regimen sensitivity.	[70]
RC	5FU, LV, OXA, Radiation	CTG	RC PDOs responses to chemoradiotherapy associated with responses in clinic. RC PDOs display the heterogeneous sensitivity to chemotherapy the same as in clinical.	[32]
RC	5-FU, CPT-11, Radiation	CTG	The sensitivity, specificity, and accuracy rates of the RC PDOs for predicting chemotherapy responses are 78.01, 91.97, and 84.43%, respectively.	[33]
PC	GEM, PTX, 5-FU, OXA, SN-38	CTG	PDOs exhibit heterogeneous responses to chemotherapy. PDO chemosensitivity profiles can mimic patient outcomes. SMAD4-deleted PC PDOs is sensitive to GEM.	[34]
PC	GEM, 5-FU, DDP, CBP, PTX, SN-38, OXA, DOC, NVB, VLB, CPT-11, CPT	CTG	Chemotherapy responses of PC PDOs indicate positive correlation with drug responses of patients in clinic.	[35]
PDAC	GEM, 5-FU, PTX, OXA, CPT-11	CTG	Pharmacotyping based on drug screening of PDCA PDOs has the potential for guiding postoperative adjuvant chemotherapeutic selection for PDCA patients undergoing surgery within the perioperative recovery period.	[71]
PDAC	FOLFIRINOX, GEM, Abraxane	MTS	PDAC PDOs display patient-specific chemotherapeutic sensitivities, and the response of PDO in vitro to FOLFIRINOX and GEM/Abraxane treatment was consistent with that of PDX in vivo.	[72]
PDAC	5-FU, DOC, doxorubicin, VP, GEM, CPT-11, MMC, OXA, PTX	Ki-67 staining	The accuracy rates of the PDOs from treatment-naive patients for predicting first-line regimens and second-line regimens are 91.1 and 80.0%, respectively. The accuracy rate of the PDOs from pretreated patients falls into 40.0%.	[36]
PDAC	Radiation	CTG	The combination of magnetic field and radiation show superior efficacy than monotherapy in PDAC PDOs.	[73]
mPC	GEM, Abraxane	CTG	The response of PDX-derived organoids and PDX models to GEM correlates with drug response in matched patients.	[57]
mPC	OXA	Organoid size	There is an excellent synergy of OXA and neoadjuvant photodynamic therapy without augment of toxicity based on mPC PDOs.	[74]
GC	DDP, OXA, 5-FU, CPT-11	CTG	Concordant cytotoxicity with chemotherapy drugs is found in GC PDOs from biopsy and surgical samples.	[42]
GC	5-FU, DDP, OXA, EPI, PTX	CTG	Common 5-FU and DDP resistances, and good OXA, EPI and PTX responses, are observed using GC PDO model.	[44]
GC	5-FU, OXA, CPT-11, EPI, DOC.	Annexin V/PI staining	An active conventional chemotherapeutic drug and a potential resistance pattern can be defined for each cancer organoid line.	[45]
GC	OXA, 5-FU, DDP, DOC, CPT-11, EPI, PTX	CCK-8	MADOs exhibit heterogeneous responses to standard-of-care chemotherapeutics.	[47]
GC	EPI, OXA,5-FU.	Live/Dead staining	PDOs of GC is useful to predict therapy response for individual patient in clinic.	[46]
GC	Nab-paclitaxel, 5-FU, EPI	CCK8	The GC PDOs is more sensitive to nab-paclitaxel than 5-FU and EPI.	[75]
PLC	Panobinostat, Ixazomib, Bortezomib, Daunorubicin, Topotecan, Plicamycin.	CTG	There used to be no approach to predict the response of human cancers to proteasome inhibitors, HDAC inhibitors, microtubule inhibitors. The drug testing based on PDO model has the potential to address the obstacles.	[48]
EC	5-FU	Organoid size	Cancer cells with high CD44 expression and autophagy are enriched in 5-FU resistance PDOs.	[51]
EADC	5-FU, EPI, DDP	CTG	The chemotherapy resistance for most EADC PDOs resembles the poor response to neo-adjuvant chemotherapy in EDAC patients.	[50]
GBC	VP	CTG	GEM-resistant and high YAP1-expressed GBC PDOs are sensitive to VP treatment.	[76]
mGIC	PTX, 5-FU, DDP	CTG	mGIC PDOs have a high accuracy value in forecasting response to chemotherapy in an individual patient.	[53]

*PDOs* Patient-derived organoids, *Ref* Reference, *CRC* Colorectal cancer, *CTG* CellTiter-Glo, *mCRC* Metastatic colorectal cancer, *RC* Rectal cancer, *GC* Gastric cancer, *PC* Pancreatic cancer, *PDAC* Pancreatic adenocarcinoma, *PLC* Primary liver cancer, *EC* Esophageal cancer, *EADC* Esophageal adenocarcinoma, *mGIC* Metastatic gastrointestinal cancer, *OXA* Oxaliplatin, *5-FU* 5-Fluorouracil, *DDP* Cisplatin, *CPT-11* Irinotecan, *PTX* Paclitaxel, *DOC* Docetaxel, *LV* Leucovorin, *GEM* Gemcitibine, *MMC* Mitomycin C, *CBP* Carboplatin, *EPI* Epirubicin, *NVB* Vinorelbine, *VLB* Vinblastine, *CPT* Camptothecin, *PI* Propidium iodide, *CAFs* Cancer-associated fibroblasts, *PDT* Photodynamic therapy, *CCK-8* Cell counting kit-8, *HIPEC* Hyperthermic intraperitoneal chemotherapy, *DPYD/DPD* Dihydrothymine dehydrogenase, *FOLFIRINOX* Oxaliplatin, leucovorin, irinotecan, 5-fluorouracil, *MTA-3* (4,5-dimethylthiazol-2-yl)-5-(3-carboxymethoxyphenyl)-2-(4-sulfophenyl)-2H-tetrazolium, inner salt, *VP* Etoposide, *MADOs* Malignant-ascites derived organoids

### Colorectal cancer (CRC)

Many groups have established biobanks of PDOs from various stages of CRC and used the CRC PDO model for radiotherapy and chemotherapy drug-screening [22, 26]. Van de Wetering and colleagues performed drug screening of 83 compounds, including chemotherapy drugs, to test the drug responses of CRC PDOs from 20 patients [22]. The accuracy of CRC PDOs in predicting the effect of radiotherapy and chemotherapy can be evaluated by comparing the drug responses between CRC PDOs and the CRC patients/PDXs. In a study comparing the drug screening of chemotherapy drugs of PDOs and their counterpart PDXs with various cancers, including CRC, the results exhibited similar chemotherapeutics drug responses between PDOs and PDXs [10]. A multicenter cohort study found that drug responses in mCRC PDOs were related to with outcome of mCRC patients [77]. The predictive value of PDOs was first demonstrated based on a living biobank of mGIC PDOs in a phase I/II clinical trial [53]. In the study, a panel of anti-tumor drugs, including those commonly used in clinic and currently in phases of clinical trials, were enrolled for testing the drug sensitivity of chemotherapy [53]. The results show that mGIC PDOs have high specificity (93%), sensitivity (100%), negative predictive value (100%) and positive predictive value (88%) in predicting chemotherapy effects in patients, suggesting that PDOs are the potential preclinical model for personalized medicine [53]. Other two similar studies have also tested the effects of chemotherapy and radiotherapy of mCRC PDOs in vitro and in vivo [31, 32]. After transplantating mCRC PDOs into immunodeficient mice, invasive CRCs and mCRCs were formed and the engrafted tumors showed the distinct sensitivity to chemotherapy, including with 5-FU, oxaliplatin (OXA), and leucovorin (LV), the same as clinical observation [32]. Wang et al. evaluated the accuracy of the organoids involving 96 samples from stage IV CRC patients in predicting chemotherapeutic responses in a blinded study [64]. The sensitivity, accuracy and specificity of the CRC PDOs for predicting chemotherapeutic responses are 63.33, 79.69 and 94.12%, respectively, indicating that the CRC PDOs can predict the drug responses of chemotherapy for individual patients [64]. Locally advanced RC PDOs can also be applied to predict chemotherapy and radiation responses of patients in clinic [33]. In the study, 96 RC PDOs from 80 patients, were treated with neoadjuvant chemotherapy (irinotecan (CPT-11) and 5-FU) and radiation in a phase III clinical trial [33]. Notably, the sensitivity, specificity and accuracy of the RC PDOs for predicting chemoradiation are 78.01, 91.97, and 84.43%, respectively [33]. Another report demonstrated that CRC PDOs can predict the clinical response for about 80% of the patients using the CPT-11-based therapy in a prospective clinical study [31]. However, the PDOs cultured in Matrigel (PDOs-^Matrigel^) fail to predict clinical response for the mCRC patients with the 5-FU plus OXA treatment (FO chemotherapeutic regimen) [31]. Instead, Xu et al. cultured the mCRC PDOs in a hydroxypropyl cellulose allyl conjugated with collagen (HA-Coll sponge) (PDOs-^Sponge^), and applied this model to assessing the effects of the FO regimen [70]. They found that the PDOs-^Sponge^ could maintain the lamin-A expression level and as their original tumor tissues, as well as maintain the feature of colorectal epithelial cells, thus successfully predicting the drug responses to the FO chemotherapeutic regimen [70]. Chemotherapy resistance is a common clinical problem, and finding credible markers and therapeutic strategies to increase chemotherapy sensitivity and even reverse chemotherapy resistance is an important application of the CRC PDOs. In a recent study, the relationship between the expression levels of different stem cell markers and the 5-FU sensitivity was explored in a cohort of CRC PDOs [78]. The results showed that the expression of Clusterin (CLU), the revival stem cell marker, was significantly increased after the treatment of 5-FU and positively correlated with drug resistance [78]. Moreover, follow up data revealed that higher CLU expression was associated with lower OS and higher recurrence rates, suggesting that CLU might be a marker of 5-FU-resistance and predicting prognosis [78]. Five PDOs were generated from resected peritoneal metastases and malignant ascites of CRC to evaluate the drug responses to OXA and mitomycin C (MMC) [69]. The results showed that OXA was less sensitive in eliminating growth of the PDOs metastasis-derived organoids than MMC, demonstrating that human peritoneal metastasis-derived organoids could be applied to explore the more effective intraperitoneal hyperthermic chemotherapy regimens for an individual patient [69]. Intra-tumoral heterogeneity is an important cause for drug resistance, and detecting drug response using several PDOs from an individual patient is a reliable way to find cancer heterogeneity and explore the best treatment option for the patients. The technologies of Single-cell RNA-Seq and drug-screening based on CRC PDOs help to find cancer heterogeneity [65]. PDOs could also be applied to detect the combined anti-cancer effect between chemotherapy and other treatment methods. Zeng et al. firstly established a library of CRC PDOs from 22 patients to evaluate the combined anti-cancer effect between hyperthermia and chemotherapy drugs [66]. They found that raltitrexed had the most significant hyperthermia synergism among the 7 common hyperthermic intraperitoneal chemotherapy drugs in CRC PDOs [66].

### Pancreatic cancer (PC)

The accuracy of PC PDOs in predicting the effect of chemotherapy can be evaluated by comparing drug responses of PDOs and those of the patients/PDXs. A PDAC patient receiving palliative chemotherapy gives an example of translational proof-of-concept [79]. PC PDOs displayed patient-specific drug sensitivities, and the model in vitro recapitulated the response to Gemcitabine (GEM)/Abraxane and FOLFIRINOX in PDX model in vivo, demonstrating that PC PDOs have potentially value in personalized medicine [72]. In another study, the response to GEM of PDOX-derived organoids and PDOX models correlated with the response of corresponding PC patients in clinic, suggesting that the PDOX-organoid platform could predict outcomes in actual patients [57]. The accuracy of the PCPDOs from treatment-naive patients for predicting first-line regimens and second-line regimens are 91.1 and 80.0%, respectively [36]. While The accuracy of the PDOs from pretreated patients falls into 40.0% [36]. PFS was much longer in treatment-naive patients treated with a predicted tumor sensitive regimen than those receiving a predicted tumor resistant regimen, indicating that patients can benefit from drug screening of PC PDOs [36]. Armstrong et al. demonstrated that the chemotherapy response curves of PDCA PDOs were reproducible, and there was difference among individual patients and in response to conventional therapies [40]. The poor survival in PDCA patients in TCGA was associated with the transcriptome of overall resistance to conventional therapies in PDCA PDOs [40]. Pharmacotyping based on drug screening of PDCA PDOs has the potential for guiding postoperative adjuvant chemotherapeutic selection for PDCA patients undergoing surgery within the perioperative recovery period [71]. Pharmacotyping profiles were also obtained from 28 PC PDOs after a median of 53 days in a prospective trial [36]. Finding credible markers to predict the response to specific chemotherapy and exploring new therapeutic strategies to increase chemotherapy sensitivity are important applications of PC PDOs. A study derived transcriptional signatures of common responders to chemotherapies using 66 PDCA PDOs [34]. PDAC patients who were most likely to have good response to chemotherapy can be predicted by the chemosensitivity-related gene signatures from the corresponding PDOs [34]. For example, PDAC patients with the OXA signature enrichment showed better drug responses to chemotherapeutic regimens than those non-sensitive patients, while PDAC patients with the 5-FU signature did not show such correlation [34]. When applied to an independent set of PDAC PDOs, this signature could correctly identify a large cohort of patients with a good outcome to that therapy [34]. Hsieh et al. reported that SMAD4 deletion was collected with a poor DFS in PDAC through bioinformatics approaches and the SMAD4-deleted PDAC PDOs was sensitive to GEM based on previous data, indicating that GEM may improve the poor DFS of PDAC patients with SMAD4 deletion [34, 80]. CDKN2A inactivation predicted poor prognosis and was associated with an upregulated estrogen response-related genes in PDAC patients, and paclitaxel (PTX) could restore the expression of CDKN2A through estrogen response in PDAC PDOs with CDKN2A inactivation, indicating that PDAC patients with CDKN2A inactivation can benefit from the PTX treatment [81]. PC PDOs are also used to detect the combined effect of chemotherapy/radiotherapy and other treatments. As observed in metastatic PC PDOs, there was an excellent synergy of OXA and neoadjuvant photodynamic therapy without augment of toxicity [74]. Nicosia et al. found that limited effect of radiation was observed in PDAC PDOs, while the combination of magnetic field and radiation showed better efficacy than monotherapy in most of the PDOs [73].

### Gastric cancer (GC)

GC PDO Biobanks have been used for chemotherapy drug screening, which show clinic response consistency, thus providing a strong basis for the selection of chemotherapy regimens for GC patients [44, 45]. Each PDO of GC displayed specific responses to epirubicin (EPI), 5-FU and OXA, and the drug responses based on PDOs correlated with the corresponding patients’ treatment effect [46]. The results indicate that GC PDOs can be applied to personlaized medicine [46]. PDOs of gastric adenocarcinoma showed distinct sensitivity for each chemotherapeutic agent, such as cisplatin (DDP), OXA, and CTP-11 [42]. GC PDOs generated from ascites exhibited distinct responses to chemotherapy, suggesting that MADOs are amenable to drug screening [47]. The results of drug-screening showed the GC PDOs is more sensitive to nab-paclitaxel than 5-FU and EPI, confirming the ambiguous role of Nab-paclitaxel [75].

### Primary liver cancer (PLC)

PDOs from tumor tissues treated with first-line therapy could be applied to screen the best possible treatment option by testing the second-line therapy. Skardal et al. tested drugs mimic to second line therapy used clinically in PLC based on PDO model [82]. A biobanking of 27 PLC PDOs was established to test the effects of anti-cancer drugs, and a rich drug response and intratumor heterogeneity was found [48]. A minority of the anti-cancer drugs including chemotherapy drugs were pan-effective, while most of the drugs appeared were either effective or ineffective only in select PLC PDO lines [48]. Notably, the heterogeneity of drug response to PLC PDOs did not correlate with the molecular signature obtaining for reduced samples [48]. In conclusion, the findings provide the basis for the studies of pan-effective drugs and personalized medicine using PLC PDOs [48].

### Esophageal cancer (EC) and biliary cancer (BC)

The poor response to chemoradiation therapy and presurgical neoadjuvant chemotherapy was associated significantly with successful formation of EC PDOs [51]. High CD44 expression and autophagy Cancer cells with are enriched in 5-FU resistance EC PDOs [51]. The advanced GBC patients had high nuclear expression of The Hippo-Yes-associated protein 1 (YAP1), and GBC patients with subserosal invasion and high expression of YAP1 had poor survival [76]. Interestingly, GEM-resistant PDOs with high expression of YAP1 from GBC patients were sensitive to VP treatment, providing a novel therapy for GEM-resistant GBC patients [76].

## GIC PDOs as an advantageous preclinical model for targeted therapy

Whole-genome sequencing (WGS) and targeted therapy usher in the era of precision cancer therapy. Unlike radiotherapy and chemotherapy, targeted therapy can select specific targeted drugs based on the specific gene mutation using WGS. However, genomic and transcriptomic profile is usually not enough to identify effective treatments for mGIC patients in clinic. Moreover, there are still many promising therapeutic targets or targeted therapy drugs that have not been authorized for the treatment for GICs. While the PDO model can more realistically reflect the therapeutic effect of targeted drugs and more efficiently promote the transformation of new targeted drugs from basic research to clinical application [10]. Besides, targeted drug resistance is the main reason for the poor prognosis, and using PDOs to find the better strategy for targeted drug therapy is another key application of tumor PDOs. Overall, for targeted therapy, the PDO technology combined with next-generation sequencing can help patients choose suitable treatment methods and find new treatment strategy to overcome clinically targeted drug-resistant problems. The precision treatments for targeted therapy using PDOs of GICs were summarize in Table 3.

**Table 3:** Precision treatment for targeted therapy using GIC PDOs

Cancer type	Target	Assay	Key findings	Ref.
CRC	EGFR, AKT, RAS, BRAF, Wnt, PI3K, IGF1R, ERBB	CTG	The activity of cetuximab in KRAS wild-type PDOs was the same as that in corresponding CRC patients. The effectiveness of Nutlin-3a was confirmed in TP53 wild-type PDOs.	[22]
CRC	MEK, mTOR, VEGFR, EGFR	CTG	Linking drug sensitivity patterns and molecular profiles with based on PDOs identify new biomarkers to predict specific drug sensitivity in CRC.	[26]
CRC	EGFR, MEK, CRAF, VEGFR, mTORC1/2, PI3K, RTK	CTG	Drug screening in multiple subpopulations of organoids from the same CRC patient helps to improve the outcome of patients in clinic for its better understanding of intra-tumoral heterogeneity in drug response.	[28]
CRC	MEK	DAPI/PI staining	MEK inhibition increased Wnt activity, and stemness- and cancer relapse- associated gene signatures, revealing a side effect of clinically used MEK inhibitors.	[83]
CRC	Hedgehog, Notch, Wnt	Alamablue	There are synergy effects between Hedgehog signal inhibitors and chemotherapy drugs used in clinic.	[84]
CRC	BTK	CTG	The combination of BTK inhibitors with 5-FU can be a treatment strategy in CRC patients.	[85]
CRC	EGFR, RAS, ERK, PI3K, AKT	CTG	There is a synergistic effect of MEK and pan-HER inhibition on mutant RAS CRC PDOs. However, the treatment induces a cell cycle arrest instead of cell death, leading to the inability of long-term effectiveness of the therapy in mutant RAS CRC patients.	[86]
CRC	MEK, EGFR, IGF1R, HDAC, PI3K, COX-2	CTG	KRAS and TP53 mutations PDOs are resistant to most drugs, except for trametinib. For APC mutation patient, EGFR inhibition is most effective strategy for CRC. The combination of HDAC inhibitors and EGFR inhibitor was more effective than the FOLFOX regimen in PDO and PDX models.	[10]
CRC	EGFR	Organoid size	The CRC PDOs knocked out of all RASGAPs are generated, only loss of NF1 leads to the activation of RAS-ERK signaling and resistance to limited EGF stimulation, suggesting that NF1-deficient CRC patients may not response to anti-EGFR therapy.	[87]
CRC	PFKFB3	Organoid size	KAN0438757, the inhibitor of glycolysis-related gene PFKFB3, may be a promising therapeutical approach for CRC.	[88]
CRC	PDGFRA, PDGFRB, FLT3	CCK-8	Crenolanib suppresses the growth of both KRAS mutation PDOs and KRAS/BRAF wild-type PDOs, suggesting that crenolanib may be applied for CRC patients.	[89]
CRC	MEK	MTS assay	The ribosomal pS6 has great value of predicting the drug response to trametinib (a MEK inhibitor) in RAS/BRAF mutant CRC PDOs.	[90]
CRC	mTOR, MNK	Cell-Titer Blue assay	PDOs with KRAS mutation sustain expression of c-MYC via the MNK/eIF4E pathway in CRC. Patients with activation of h mTORC1 and MNKs may benefit from a c-MYC-dependent co-targeting strategy in clinic.	[91]
CRC	PI3K/mTOR, CDK4/6, VEGFR1, VEGFR2, VEGFR3, PDGFR-β, c-Kit, Smoothened, EGFR, AKT, MEK	CTG	Nineteen out of 25 CRC PDOs show good responses to one or more drugs. However, CRC patients treated with the recommended treatment based on the drug screening pf PDOs do not exhibit good outcome.	[92]
CRC	EGFR, RAF	Organoid size	EGFR activited MAPK signaling in KRAS/BRAF mutant CRC PDOs, providing a mechanism of the effectivity of EGFR inhibition within combination therapies against BRAF/KRAS mutant CRC.	[93]
CRC	ERK	CTG	The molecular signature of human original CRC tissues may represent the drug responses in the CRC PDOs, but is not completely overlapping.	[94]
mCRC	PARP	CTG	The organoids from patients with limited therapeutic options and poor prognosis is sensitive to the PARP inhibitors.	[30]
mCRC	EGFR, MDM2, TP53, CDK, MEK, BRAF, mTOR, AKT	CTG	Three drug response clusters are identified based on the sensitivities to MDM2 and EGFR inhibition. The combination of MEK and mTOR/AKT inhibition may be a potential strategy for CRC patients with the MDR profile and a RAS mutant background.	[67]
RC	EGFR	CTG	KRAS-mutant CRC PDOs are resistant to cetuximab, while the KRAS-wild-type PDOs are sensitive to cetuximab.	[32]
PC	FGFR, MEK, mTOR	CTG	Targeted therapy sensitivities based on the PDO pharmacotyping may improve the personalized medicine for the patients with PC.	[34]
PC	AURKA, PIK3CA, HER2, EGFR, AKT, PRMT5	CTG	Therapeutic response to targeted drugs shows heterogeneity in PC PDOs.	[35]
PDAC	EZH2	CTG	Organoids from different patients with PDAC show distinct responses to the EZH2 inhibitors, which associated with H3K27me3 in PDOs and corresponding patient tumor.	[38]
PDCA	SHP2, MEK	Tumor volume	Synergy effect is observed between SHP2 inhibitor and MEK inhibitor in PDCA PDOs, indicating the dual MEK/SHP2 inhibition may be a promising targeted therapy for KRAS-mutant patients.	[95]
PC	ATR, WEE1	CTG	DDR deficiency and high replication stress are independently of each other, providing therapy strategy for DDR proficient and high replication stress PC patients with by WEE1 or ATR inhibition.	[96]
PDCA	MEK, AKT, EGFR, ERBB	CTG, tumor volume	Dual of MEK/AKT inhibition is synergistic with ERBB inhibition, and the combination of MEK antagonists with a ERBB inhibitor shows the highest activity in PDCA PDOs.	[97]
PDCA	MEK, HSP-90	CTG	The inhibition of HSP-90 increases the anti-cancer activity of MEK inhibition in PDOX model by overcoming the compensatory activation of resistance pathways induced by MEK inhibition.	[98]
PDAC	DCLK1	CTG	DCLK1-IN-1, the first selective probe of the DCLK1 kinase domain, shows anti-cancer activity in PDAC PDOs by modulating cell motility related proteins.	[99]
GC	STAT3, VEGFR, ATR, PARP, SMO, EGFR, ARID1A, CDK4/6, MEK, RAF, PI3K, mTOR, HER2, HGFR, WNT, BCR, CDK, TNF-a, TTK, PLK	CTG	The GC PDOs shows good responses to some new target drugs and some target drugs currently in clinical trials. Besides, drug response heterogeneity is found in different PDOs from the same GC patient.	[44]
GC	HER2, ERBB2, c-KIT, CDK4/6	Annexin V/PI	The mutational features of GC PDOs allow the palbociclib treatment for CDKN2A loss and the trastuzumab treatment for ERBB2 alterations.	[45]
GC	TrxR	Organized size	Ethaselen (a TrxR inhibitor) inhibits the growth of GC PDOs, indicating that the ethaselen could be an effective drug for the treatment of GC.	[100]
GC	PI3K-AKT	Ki-67 staining	The PI3K-AKT pathway protects FOXO3-Cyt GCs from FOXO3-mediated growth suppression and an AKT inhibitor suppresses the proliferation of FOXO3-Cyt GC PDOs, indicating that the targeting the PI3K-AKT pathway may have potential applications for FOXO3-Cyt GC treatment.	[101]
PLC	RTK, MAPK, PI3K, AKT, mTOR	CTG	SCH772984, the ERK inhibitor, may be a promising treatment for PLC based on the PLC-derived organoids.	[25]
HCC	Hedgehog, RAF	CTG	GANT61 (a Hedgehog signaling inhibitor) reverses the resistance of sorafenib in CD44(+) HCC PDOs.	[102]
HCC	FAO	Numbers of organoids	The inhibition of FAO by Eto in HCC PDOs with CPS1-deficiency shows good response.	[103]
HCC	Omacetaxine	CTG	Omacetaxine is found to be one of the most effective drugs in HCC PDOs and the effects were confirmed using a cohort of 40 HCC PDOs.	[104]
EADC	PI3K, IGF1R, EGFR, MDM2, ERK, MEK1/2	CTG	The EADC PDO model serves as a reliable pre-clinical tool for personalized medicine.	[50]
BC	MDM2, EGFR, mTOR	CCK-8	Drug sensitivity is associated with genomic profiles in BC PDOs, and they can complement each other in precision medicine.	[52]
PBC	ILK	AlamarBlue Cell Viability	An ILK inhibitor suppresses the proliferation of PBC PDOs.	[59]
GIC	BRAF, EGFR, AKT, ERBB2, PI3K, mTOR, CDK4/6	CTG	There is high specificity (93%), sensitivity (100%), negative predictive value (100%) and positive predictive value (88%) of GIC PDOs in predicting response to targeted drugs for patients in clinic.	[53]
aGEA	EGFR	NM	In EGFR-amplified aGEA PDOs, the EGFR inhibitors even antagonize the effects of EPI.	[105]

*PDOs* Patient-derived organoids, *Ref* Reference, *CRC* Colorectal cancer, *CTG* CellTiter-Glo, *mCRC* Metastatic colorectal cancer, *RC* Rectal cancer, *GC* Gastric cancer, *FOXO3-Cyt* FOXO3 cytoplasmic distributed, *PC* Pancreatic cancer, *PDAC* Pancreatic adenocarcinoma, *PLC* Primary liver cancer, *HCC* Hepatocellular carcinoma, *CPS1* Carbamoyl phosphate synthetase I, *FAO* Fatty acid β-oxidation, *Eto* Etomoxir, *GBC* Gallbladder cancer, *YAP1* The Hippo-Yes-associated protein 1, *EADC* Esophageal adenocarcinoma, *BC* Biliary cancer, *PBC* Pancreato-biliary cancers, *ILK* Integrin-linked kinase, *MDR* Multi-drug resistance, *GIC* Gastrointestinal cancer, *aGEA* Advanced gastro-oesophageal adenocarcinoma, *HSP* Heat shock protein

### Colorectal cancer (CRC)

There may be three types of targeted drugs in CRC: anti-EGFR antibodies such as panitumumab and cetuximab; anti-VEGF like such as bevacizumab, ramucirumab and aflibercept; multikinase inhibitors such as regorafenib [106]. The accuracy of PDOs in predicting the drug responses of targeted therapy should be evaluated by comparing drug response results of PDOs and those of the patients/PDXs. In a study comparing GIC PDOs and their corresponding PDXs from various cancers, including CRC, there were mimic drug responses of the FDA-approved targeted drugs between PDOs and PDXs [10]. The BRAFV600E mutation CRC PDOs showed great reduced cell viability instead of promoting cell apoptosis after the treatment of vemurafenib (a BRAF inhibitor) when compared with those of the wide type BRAF CRC PDOs, which may explain the ineffectiveness of BRAF inhibitors for mCRC patients in clinic [53]. CRC PDO-X model was developed to explore whether the drug responses to regorafenib was consistence in PDOs and in patients [53]. PDO-Xs derived from a patient sensitive to regorafenib was sensitive to regorafenib as well, whereas PDO-Xs derived from a patient resistant to regorafenib was resistant to regorafenib, too [53]. Meanwhile, pre-treatment and post-treatment PDO-Xs were established from a patient with mCRC, the results showed that the pre-treatment PDO-Xs were sensitive to regorafenib and the post-treatment PDO-Xs were resistant to regorafenib, indicating that CRC PDOs can capture acquired resistance to regorafenib [53]. A multicenter cohort study showed that drug responses in mCRC PDOs were consistent with outcome of patients [77]. However, Ooft et al. insisted that the drug screening of PDO technology has limited value in personalized medicine [92]. They organized a single-center, single-arm and prospective intervention SENSOR trial to assess the value of PDOs for the treatment with investigational or off-label drugs, and found that the recommended treatment based on PDOs did not show an objective response for the patients [92]. CRC PDO model can be used for optimizing therapeutic choices for the individual patient by comparing different treatment programs. The drug screening on APC mutation CRC PDOs suggested that the growth inhibition of cancer cells was greater in the combination of histone deacetylase (HDAC) inhibitors and afatinib group than that of FOLFOX regimen group [10]. Verissimo et al. established a living biobank of CRC PDOs to test the anti-cancer effects of different RAS pathway inhibitors in a preclinical setting [86]. The result showed Bcl-2 inhibition could overcome resistance to MEK and pan-HER inhibitors in RAS-mutant CRC PDOs and PDXs [86]. The study demonstrates the value of CRC PDOs in accessing drug responses in a preclinical setting [86]. Although the developing targeted drugs improve the PFS of CRC patients, primary and secondary resistance to the current targeted therapy remains an urgent clinical problem. Applying CRC PDO model to identify other targetable pathways and novel biomarkers is of great importance to improve OS for these primary or secondary resistant patients. High-throughput screening of a panel of targeted therapy agents CRC PDOs was done, and it was observed that TP53-mutation organoids were insensitive to nutlin-3a (MDM2/TP53 inhibitor), and KRAS-mutant organoids were resistant to the cetuximab and afatinib (the EGFR inhibitors) [22]. In addition, they suggested the Wnt secretion inhibitors as a novel treatment strategy for the RNF43 mutant CRC patients based on drug screening of CRC organoids [22]. Pharmacogenomic profiling of mCRC PDOs was done by performing the genomic profiling and drug sensitivity screening [67]. There are three drug response clusters identified based on sensitivities to MDM2 and/or EGFR inhibitors, and corresponding with RAS mutations and TP53 activity [67]. Potentially effective therapies could be nominated for 18 patients using the model [67]. Glycolysis is one of the hallmarks of cancer and targeting glycolysis may be the novel therapeutical strategy for CRC [88]. KAN0438757, the inhibitor of glycolysis-related gene PFKFB3, showed a significant anti-tumor effect in PDOs of CRC, but had no cytotoxicity in normal colonic organoids, indicating a promising therapeutical approach for CRC [88]. Signorile et al. showed that the expression of p38α in locally advanced CRC stem cells (CRC-SCs) was from patients reduced, and advanced CRC patients with high p38α levels had reduced DFS and PFS [107]. Ralimetinib (the p38α kinase inhibitor) made the CRC-SCs from patients more sensitive to chemotherapy, and the combination of ralimetinib with trametinib (the MEK1 inhibitor) showed a synthetic lethality effect, suggesting that p38α targeting in CRC-SCs may be a novel CRC treatment strategy for CRC [107]. BRAF or RAS mutations are connected with bad prognosis in CRC. Although the inhibitors of ERK and MEK are effective in the BRAF or KRAS mutational cells, the drug response in clinic is not always good. Using RAS/BRAF mutant CRC PDOs may help to find out the reasons for unexpected therapeutic effect of MEK/ERK inhibitors, and find biomarkers to predict the therapeutic effect of the inhibitors, and look for new treatment strategy for the refractory CRC. Tayama et al. reported that 5/6 cases of KRAS and BRAF wild-types were resistant in CRC PDOs, while 6/7 cases with either KRAS or BRAF mutations showed good drug response to SCH772984 (an ERK inhibitor), suggesting that the molecular signature of human original CRC tissues may largely resemble the drug sensitivity in the PDOs but is not completely overlapping [94]. Drug screening of PDOs and gene sequencing may complement each other to guide the personalized medicine for cancer [94]. MEK inhibition could lead to increased LGR5 levels, Wnt activity and stemness- and cancer relapse-related gene expression in CRC PDOs, revealing a side effect of MEK inhibition via inducing stem cell plasticity [83]. The ribosomal protein S6 (pS6) had great value in predicting the treatment effects of trametinib (a MEK inhibitor) in RAS/BRAF mutant patients with CRC [90]. Crenolanib, targeting tyrosine kinase receptors, including PDGFRA, PDGFRB, and FLT3, suppressed the growth of both KRAS/BRAF mutation PDOs and KRAS/BRAF wild-type PDO, suggesting that crenolanib may be applied for CRC patients [89]. The combination of mTOR/AKT and MEK inhibition may be a potential strategy for CRC patients with the multi-drug resistance profile and a RAS mutant background [67]. Knight et al. showed that KRAS with G12D mutation PDOs sustain expression of c-MYC via the MNK/eIF4E signaling in CRC [91]. Patients with high signaling through the MNKs and mTORC1 may benefit from a c-MYC-dependent co-targeting strategy in clinic [91]. Ponsioen et al. demonstrated that EGFR activity activated MAPK signaling in BRAF/KRAS mutant CRC PDOs, providing a mechanism of the validity of EGFR inhibition within combination treatment for KRAS/BRAF mutant CRC patients [93]. However, anti-EGFR monotherapy is not suitable to all RAS-mutant CRC. The CRC PDOs knocked out of all RASGAPs were generated using CRISPR technology, only the NF1 deficiency led to improved tolerance to limited EGF stimulation and enhanced activation of RAS-ERK signaling, suggesting that the loss of NF1 in CRCs may not response to anti-EGFR therapy [87]. Schumacher et al. applied the technology of drug screening of organoids from multiple subpopulations of the same CRC patient to study the intra-tumoral heterogeneity in drug response [28]. It was observed that MAPK signaling showed unexpected heterogeneity in CRC PDOs and was associated with drug response heterogeneity to EGFR inhibition, implying that drug testing in multiple subpopulations of the same patient may improve the PDO-based drug response prediction [28]. The heterogeneity of patients with CRC liver metastases was explained by another team [67]. The study demonstrated that there was little intra-patient drug sensitivity heterogeneity among organoids from multiple liver metastases of ten patients with mCRC, indicating that drug screening using PDOs may provide novel treatment selection for mCRC [67].

### Pancreatic cancer (PC)

Recently, targeted therapies, such as PARP inhibitors targeting BRCA1 or BRCA2 mutations, and TRK inhibitors targeting NTRK1/2/3 fusions, have been used in PDAC patients [108]. However, only a few PDAC patients can benefit from genetic test-based therapies [108]. The combination of gene sequencing and drug screening based on PDOs can push the precision treatment of PC one step further. PC PDOs can be applied to find novel therapeutics to target PC cells and new biomarkers to predict targeted therapy effects. The drug screening of target drugs identified sensitivities untapped in clinic and underlined the value of PC PDOs for personalized medicine [35]. For example, EZP015556 (the PRMT5 inhibitor) was effective for MTAP (−) tumors and a subset of MTAP (+) tumors [35]. The value of oncogene doublecortin like kinase 1 (DCLK1) in PDAC as a therapeutic target is largely unknown. Fleur M. et al. developed DCLK1-IN-1, the first in vivo-compatible and selective chemical probe of the DCLK1 kinase domain, which showed anti-cancer activity by regulating cell motility associated proteins and signaling in PDAC PDOs [99]. Dreyer et al. demonstrated that a signature of replication stress could predict drug response to WEE1 and ATR inhibition in PC PDOs [96]. DNA damage response (DDR) deficiency and high replication stress are independently of each other in PC, offering therapy strategy for DDR proficient and high replication stress patients with PC with by WEE1 or ATR inhibition based on the drug response in PC PDOs [96]. Ras is the most frequently mutated gene in PDCA and Ras mutations are associated with poor prognosis. MEK/ERK/c-Myc, PI3K-AKT are RAS effector pathways, but combined MEK and PI3K inhibition do not exhibit effectiveness for PDAC in clinic. Using PC PDOs may help to find out new treatment strategy for the refractory PCs. Dual of MEK/AKT inhibition accompanied by increased phosphorylation of ERBB2/3 is synergistic with ERBB inhibition, and the combination of MEK antagonists with a ERBB inhibitor shows the highest activity in PDCA PDOs [97]. SHP2 activation was important resistance mechanism for blockade of MEK in KRAS-mutant cancer, and there were synergy effects between SHP2 and MEK inhibitions in PDOs of PDAC, indicating that the dual SHP2/MEK inhibitors may be applied to the treatment for KRAS-mutant PDAC patients [95]. The inhibition of heat shock protein (HSP)-90 increases the anti-cancer activity of MEK inhibition in PDOX model by overcoming the compensatory activation of resistance pathways, such as PI3K/AKT/mTOR signaling, induced by MEK inhibition [98]. Both NHWD-870 and JQ1(the inhibitors of c-MYC transcription) were efficient in MYC-high samples using PDAC PDOs, while NHWD-870 was the more effective, indicating that the combination of the molecular signatures and drug screening of PDAC PDOs could be applied to find optimal therapy for each patient in a clinical timeframe [109].

### Gastric cancer (GC)

GC, with obvious molecular heterogeneity, displays treatment resistance and aggressive behavior. Therefore, good models that keep the intra-tumoral heterogeneity are urgently needed for the personalized medicine for GC. Here, a GC PDO biobank retaining regional heterogeneity and drug response heterogeneity was constructed [44]. The GC PDOs shows good responses to some novel target drugs, including napabucasin (the STAT3 inhibitor) and abemaciclib (a CDK4/6 inhibitor), and to some target drugs currently in clinical trials, such as vistusertib and VE-822 (an ATR inhibitor) [44]. Seidlitz and colleagues demonstrated that GC PDOs can be applied to test the drug responses of known and unknown mutation-targeted drugs for the individual patient [45]. For examples, the mutational features of GC PDOs allow the palbociclib treatment for CDKN2A loss, the trastuzumab (the HER2 inhibitor) treatment for ERBB2 alterations, and the imatinib treatment for an unknown mutation of the KIT receptor in GC [45]. Moreover, ethaselen (a TrxR inhibitor) effectively regulated cell proliferation and apoptosis in GC, and was further confirmed in GC PDOs, indicating that the ethaselen may be effective for the therapy of patients with GC [100]. Tsuji et al. report that FOXO3 is a potential tumor suppressor for FOXO3 cytoplasmic distributed (FOXO3-Cyt) GC cells, while PI3K/AKT pathway activation protects FOXO3-Cyt GC cells from FOXO3-mediated growth suppression by the FOXO3 nuclear export [101]. The AKT inhibition significantly suppressed the cell proliferation of FOXO3-Cyt GC PDOs, indicating that targeting the PI3K/AKT signaling and nuclear translocation of FOXO3 may be the potential treatment for FOXO3-Cyt GC [101]. Smyth et al. explored the connection between the outcome of patients and EGFR copy number (CN) in a random, first-line, phase III clinical trial of chemotherapy in combination with panitumumab (the anti-EGFR monoclonal antibody) in advanced gastro-oesophageal adenocarcinoma (aGEA) [105]. EGFR amplification connected with poor survival in the intention-to-treat patients [105]. Surprisingly, EGFR inhibition plus chemotherapy did not improve the survival of EGFR CN gain patients, and the combination of EGFR inhibitors and EPI even resulted in increased viability in EGFR-amplified aGEA PDOs [105]. Taken together, EGFR inhibitors may antagonize the anti-tumor effect of anthracycline chemotherapy drugs for aGEA [105].

### Primary liver cancer (PLC)

Sorafenib and lenvatinib are the targeted therapies approved for use as first-line treatment for HCC, the most common type of PLC. However, HCC patients show heterogeneity in response to sorafenib and lenvatinib in clinic. HCC PDOs can be applied to predict targeted agent sensitivities for individual patient and find novel therapy for resistant patients. The HCC PDOs displayed sorafenib treatment heterogeneity in different patients, implying the great value of HCC PDOs to predict targeted agent sensitivities for individual patient [49]. CD44 (+) HCC PDOs were resistant to sorafenib by upregulation the expression level of CD44 and Hedgehog signaling [102]. GANT61, a Hedgehog signaling inhibitor, could increase sorafenib sensitivity through inhibiting the expression level of CD44 and Hedgehog signaling in CD44(+) HCC PDOs [102]. The results imply that the combination of Hedgehog pathway inhibition and sorafenib may be the effective therapy for CD44(+) HCC patients [102]. Huch M and colleagues successfully performed a panel of anti-cancer drugs screening of PLC PDOs [25]. The results indicate that the PLC PDO platform can be applied to personalized medicine for individual patient, and among them, SCH772984 (the ERK inhibitor) may be a promising treatment for PLC patients [25]. Wu et al. identified that carbamoyl phosphate synthetase I (CPS1)-deficient HCC patients had poor clinical prognosis, and the liver-specific urea cycle (UC) was downregulated in HCC [103]. The downregulation of UC slowed down of the tricarboxylic acid cycle, while CPS1 deficiency caused excess ammonia, which activated fatty acid β-oxidation (FAO) through p-AMPK [103]. Blocking FAO by Eto provides benefit for CPS1-deficiency HCC PDOs [103]. Omacetaxine is found to be one of the most effective drugs in HCC PDOs and the effects were confirmed using a cohort of 40 HCC PDOs [104]. Omacetaxine inhibited overall protein synthesis and key oncogenes, such as PLK1, was identified as a molecular target for omacetaxine [104]. Further clinical trials should be done to evaluate the therapeutic effects of omacetaxine for HCC patients.

### Esophageal cancer (EC) and biliary cancer (BC)

Medium-throughput drug sensitivity testing based on PDOs demonstrate the potential value of inhibition of receptor tyrosine kinases and downstream mediators in treating EDCA patients, highlighting the important role of EDCA PDOs in precision medicine [50]. PDOs act as an pivotal preclinical model for exploring gene-drug connection in BC. Saito Y et al. demonstrated that the wild-type TP53 mutant BC PDOs were very sensitive to nutlin-3a, while the TP53 mutant PDOs were resistant to nutlin-3a [52]. SOX2 may applied to predict the outcome for patients with BC based on the genomic profiling of PDOs [52]. Taken together, PDOs may be a powerful preclinical model for the identification of therapeutic drugs and prognostic biomarkers for BC [52]. Shiihara et al. performed exome sequencing of PBC PDOs and paired tumor tissues, and found the shared aberrations may be the candidates for targeted therapies, such as integrin-linked kinase (ILK), which was further confirmed in PDOs [59]. The combination of genomic profiling and PDO model allowed the identification of genotype-oriented targets and gave a proof-of-concept approach to personalized medicine for patients with PBC [59].

## GIC PDOs as an advantageous preclinical model for immunotherapy

Immunotherapy is a novel treatment that invites the patient’s immune system to kill tumors. That cancer cells exhibiting enough immunogenicity to trigger immune response is essential for immunotherapy [110, 111]. Mutational status of malignant cells to product neo-antigens is in charge of immune response [111, 112]. TME affected drug response to cancer. However, it is hard to characterize TME for it is challenging to maintain viability in human tissue in vitro culture. The PDO organoid in vitro and PDO-X in vivo can serve as the reliable models to study the effects of specific genetic mutations on tumor behavior and TME. Several studies apply the organoid technology to the immunotherapy, as exemplified by the co-culture of intraepithelial lymphocytes with mouse intestinal organoids at the addition of IL-2/7/15 in the culture medium [113]. The findings imply that T lymphocytes from healthy people could be co-cultured with organoid culture, demonstrating the possibility of using PDOs of cancer to study the effects of T lymphocytes. Neal JT et al. indicate that the patient-derived tumor organoids using the air-liquid interface (ALI) technology can reserve the intrinsic tumor T-cell receptor profile and anti-PD-1/PD-L1-dependent human tumor-infiltrating lymphocyte (TIL) activation [114]. The co-culture of HCC PDOs with fibroblasts or endothelial cells increased the expression of epithelial-mesenchymal transition (MMP9 and TGF-β), inflammation (TNF and CXCL12) and neo-angiogenesis (VEGFR and HIF-α) [115]. Immunotherapy has shown clinical benefit in anti-tumor immune responses, but most patients in clinic are not responsive to immunotherapy due to the heterogeneity of human leukocyte antigen (HLA) and T cells from patient-specific neoantigens [116–118]. Dijkstra KK and colleagues successfully achieved the co-culture of the patient’s peripheral blood lymphocytes (PBLs) with tumor organoids, and they found that the enriched tumor-reactive T cells with patient-specific immunogenic mutations could identify and kill the tumor cells in PDOs [119]. The results show that cancer organoid culture is a promising way to enrich tumor-reactive T cells and to predict response to immunotherapy for patients with cancer. Overall, the research of immunotherapy based on PDOs of GICs is still on its preliminary stage, and PDOs of GICs co-cultured with immune cells may help to predict therapeutic effects and study new therapeutic strategies for immunotherapy. The precision treatments for immunotherapy using GIC PDOs of were summarize in Table 4.

**Table 4:** Precision treatment for immunotherapy using GIC PDOs

Cancer type	Immunotherapy	Assay	Key findings	Ref.
CRC	IFNγ treatment	FC	Only 3/612 non-silent mutations encode for neoantigens that are detectable by MS, establishing a low detection rate for non-silent mutations encoding for presented neoantigens. The finding may partly explain the unsatisfactory effect of ICIs for patients with non-hypermutated CRC.	[120]
CRC	CEA and CD3	FC	Heterogeneity of CEA expression contributed to low response to cibisatamab in CRC PDOs.	[121]
CRC	CAR-engineered lymphocytes	Organoid numbers	The CRC PDO platform to access tumor specificity and CAR efficacy and was established.	[122]
GC	PD-1 blocking antibody	Organoid areas	The co-culture of GC PDOs and immune cells may be used to study the function of MDSCs within the TME. The mTOR signaling regulates PD-L1 expression induced by GLI in GC	[123]
PDAC	anti-PD-1 and GEM	Apoptosis assay	The combination of GEM with anti-PD-1 induces sustained relief or even the complete elimination of aggressive PDAC by targeting Pin1.	[124]

*PDOs* Patient-dervied organoids, *Ref* Reference, *CRC* Colorectal cancer, *FC* Flow cytometry, *MS* Mass spectrometry, *ICI* Immune checkpoint inhibitor, *CAR* Chimeric antigen receptor, *MDSCs* Myeloid-derived suppressor cells, *TME* Tumor microenvironment, *PDAC* Pancreatic ductal carcinoma, *GEM* Gemcitabine

### Colorectal cancer (CRC)

Co-cultures of tumor PDOs with high mutational burden with PBLs can generate CD8 (+) T cell clones with the presence of putative neoantigens [119]. In theory, such co-cultures could enrich effector T cells for adoptive cell transplantation or enhance the response of effector T cells to the specific patient’s cancer cells [119]. Cho et al. explored the TME of CRC PDOs and found that CRC PDOs showed cancer-specific immune-related gene heterogeneity [125]. For example, HLA-II expressed CRC PDOs were associated with good outcome in clinic and present a subgroup of patients with Intrinsically Immunogenic Properties (Ca-IIP) and immune stimulation cells [125]. The Ca-IIP phenotype with low intrinsic E2F/MYC signaling expression was associated with favorable prognosis [125]. While the TME phenotype with microsatellite instability, APC/KRAS mutations, and active Wnt/β-catenin signaling pathway was connected with poor prognosis [125]. These findings may help to find optimal immunotherapy for individual patient using the PDO-based patient stratification [125]. Schnalzger et al. established the CRC PDOs to study CAR-Chimeric antigen receptor (CAR) efficacy for the indivadul patient [122]. They confirmed the validity of CAR-engineered NK-92 cells directed targeting EPCAM in different CRC PDOs [122]. The tumor antigen-specific cytotoxicity of CAR-NK-92 cells targeting CRC PDOs expressing EGFRvIII and FRIZZLED receptors was also tested [122]. The heterogeneity of CEA expression contributed to insensitive to cibisatamab (the anti-CEA and anti-CD3 antibody) in the T cell and CRC PDO co-culture systems [121]. The combination of cibisatamab and the WNT/β-catenin inhibition may be a potential strategy to increase drug sensitivity to cibisatamab for CRC patients, making it possible for the co-culture model to find novel prognostic biomarkers and new strategy to increase sensitivity to immunotherapy in the clinic [121]. Xu et al. identified that atractylenolide I (ATT-I) could strengthen T cell-mediated cytotoxicity in both human CRC cells and PDOs [126]. Combination of ATT-I with the proteasome 26S subunit non-ATPase 4 (PSMD4) enhanced the antigen-processing activity of immunoproteasome, thereby augmenting MHC-I-mediated antigen presentation on CRC cells [126]. Collectively, targeting the immunoproteasome with ATT-I enhances promotes cancer cell antigen presentation and the cytotoxicity of effector T cells, thus strengthening the efficacy of immunotherapy [126].

### Gastric cancer (GC) and pancreatic cancer (PC)

The co-cultures of immune cells and PDOs revealed that PD-L1-expressing PDOs were resistant to nivolumab (a PD-1 blocking antibody) in the presence of PMN-MDSCs and were sensitive to anti-PD-1/PD-L1 drugs with the depletion of PMN-MDSCs [123]. Moreover, rapamycin (the mTOR inhibitor) could inhibite the expression of PD-L1 though GLI1 and GLI2 in the co-culture system [123]. Taken together, the immune cells and PDO co-culture model may be applied to study immunosuppressive activity of MDSCs within the TME of GC, and to find the mechanisms regulating PDL1 expression in GC [123]. PDOs can also be applied in the combination therapies such as immunochemotherapy for GIC patients. Koikawa et al. demonstrated combination of GEM with anti-PD-1 induced sustained relief or even the complete elimination of aggressive PDAC by targeting Pin1 [124]. Organoid apoptosis method was used to detect the anti-cancer effects of Pin1 inhibition on immunotherapy or the combination of immunotherapy and chemotherapy in the co-culture system of PDAC PDOs and human primary peripheral blood mononuclear cells (PBMCs) [124]. The results revealed that the Pin1 inhibition increased both the anti-PD1−/anti-PDL1- induced organoid apoptosis and the GEM + anti-PD1−/anti-PDL1-induced organoid apoptosis of PDAC [124].

## Summary of the application of GIC PDOs in personalized medicine

Besides these therapies, applying human tumor organoids to detect the response to oncolytic adenovirus (OA) therapy, the novel anti-cancer treatment, has also been explored. The responses of human tumor organoids to a panel of OAs show heterogeneity in cytotoxicity and in synergism with standard chemotherapy for individual patients [127]. OA cytotoxicity in human tumor organoids was able to predict the anti-tumor efficacy of OAs in vivo in both primary tumors and metastatic foci [127]. Overall, GIC PDOs have shown diverse responses to chemotherapy, radiotherapy, targeted therapy, immunotherapy or combination therapy. In current cohorts of patients, the response of GIC PDOs to anti-cancer therapies resembles the response of the patients in clinic [10, 34, 53]. For radiotherapy and chemotherapy, which have a narrow therapeutic index in vivo, the drug responses of GIC PDOs to these therapies may reflect the real responses of corresponding patients in clinic [10, 34, 53]. In terms of targeted therapy, the PDO-based drug susceptibility testing and genetic testing-based drug response prediction can complement each other, thus putting the personalized medicine for GICs forward. To immunotherapy, the successful establishment of co-cultures of GIC PDOs and immune cells provides a novel and promising way to predict immunotherapy response in clinic. Additionally, GIC PDOs exhibit prospects in new drug development, and clinical trials involving PDOs can help to determine whether GIC PDOs may predict their response to therapies with high accuracy, help to choose the optimal therapy for individual patients, and find novel therapy to reverse drug resistance. However, it is worth noting that the PDO is an in vitro preclinical model, it may fail to predict outcome for treatment with the drugs, whose active ingredients can only be released after being metabolized in vivo. For example, CRC PDOs are inefficient to predict the drug responses to 5-FU alone or combined with OXA [26, 31]. For 5-FU is catabolized via the DPYD/DPD in vivo, and only 1–3% of the 5-FU concentration leads to active metabolites in plasma, suggesting that anabolic routes might be less efficient in vitro [26]. Besides, Beutel et al. demonstrate that the CRC PDO model displays a high response prediction rate in treatment-naive patients but fail to predict the chemotherapy response in pretreated patients, making drug screening of PDOs questionable in predicting drug response for pretreated patients [36]. Therefore, deep understanding the advantages and limitations of the GIC PDO model, defining the scope of application of the GIC PDOs in predicting the treatment effect of patients, and combining with the gene sequencing technology, will help to play the greatest role of GIC PDOs in personalized medicine.

## Limitations

The present version of the model is still immature and imperfect. There are still several problems should to be solve to reform the clinically relevant PDOs. First of all, the “tissues in a dish” GIC PDO model only comprising epithelial layer without TME can not 100% copy the structural and functional features of human cancers [128]. The shortage restricts the accuracy of GIC PDOs testing the sensitivity of stromal targeted drugs and immunotherapy drugs. Supplementing GIC PDOs with other human cell type in an improved culture medium, which can preserve most or even all cell types, may solve the problem. For example, the co-culture of PDAC PDOs with patient-derived cancer associated fibroblasts (CAFs), which can provide WNT ligands for PDAC, is capable of evaluating drug sensitivity, making the co-culture model a potential way to guide personalized medicine [21, 129]. Secondly, being unable to obtain pure tumor organoids is another key problem for the establishment of GIC PDO model, which can further affect its application in the precision medicine. For the higher rates of mitotic failures and cell death in cancer organoids, may human tumor organoids grow even slower than the normal tissue organoids, resulting in growth of cancer organoids with normal epithelial organoids [86, 130]. GIC PDOs can be overgrown by normal organoids derived from healthy intestinal [131], the liver [25] and pancreas [21] tissues. For GC, some teams insist that the tumor organoids can actually be overgrown by gastric epithelial tissue-derived organoids [43, 44], others demonstrate that the GC organoids grow much faster than gastric normal cell-derived organoids [45]. It is necessary to either grow the tumor tissues under selective culture conditions or use pure tumor material for the establishment of pure tumor organoid. One commonly used solution to this issue is to select cancer cells that carry the most frequent mutations in the corresponding cancers, such as KRAS for PDAC organoids [21], Wnt and R-spondins for CRC organoids [15, 132], TP53 for GC organoids [43, 44]. Cancer cells harboring EGFR and downstream effector mutations in the signaling can be selected by EGF withdrawal [23, 130, 133]. However, the selection would induce intra-tumoral heterogeneity lost, or might induce novel mutations mission after the long-term culture. Using pure tumor cells as starting material and choosing the tumor cells by microscopical selection can be another strategy [44]. Thirdly, the culture conditions of PDOs lack uniformity. The culture conditions of PDOs may be different for different cancer types, different pathological types, and different genotypes. In some cases, even the culture conditions of PDOs of same cancer types and same pathological types may be different in different research without explanation. The impact of these differences on the success rates of PDOs and the drug effectiveness needs to be further explored. Moreover, there is still a large need to increase the establishment success rates and to decrease the generation time of cancer PDOs from both surgical and biopsy specimens. One of the major challenges in using GIC PDOs in personalized treatment is the time required from biopsy/surgery to the functional characterization. Zahra Dantes et al. try to speed up the genetic prolifing of PDOs by testing the cell-free DNA from conditioned media of individual PC PDOs to detect gene mutations early on during the expansion procedure [37]. The results show that the mutational profile of the PDO supernatant recapitulate the genetic alterations of the human original tumor tissues, indicating feasibility of this method to detect drug response on PDOs in a reduced time frame [37]. Gao et al. demonstrate that single-cells of PDOs are accurate for fast drug testing in GICs, and using early passage PDO single-cells for drug screening decreases time from tumor organoid establishment to appliaction in clinic [134]. Last but not the least, biomaterials used to generate the PDOs are mouse-derived reconstituted extracellular matrix (ECM) hydrogels, such as basement membrane extract (BME) and Matrigel. The uncleared defined composition and immunogenicity of ECM hydrogels will affect the applications of GIC PDOs in personalized medicine. Antonius Chrisnandy et al. reported a family of well-defined synthetic hydrogels that could promote organoid generation via reversible hydrogen bonding-mediated dynamic rearrangements [135]. The stress-relaxing matrices could promote crypt budding in intestinal stem-cells by forming Paneth cell and increasing symmetry breaking [135]. Such well-defined and stable synthetic hydrogels allow the generation of intestinal organoids, and further optimizations need to be done on the basis of currently hydrogels to achieve the successfully establishment of GIC PDOs.

## Conclusion

Despite the limitations of the imperfect GIC PDO model at present. The promising technology of GIC PDO culture retain the molecular, cellular, physiological, histological features, biological behaviors, and intra-tumoral heterogeneity of human original tumor tissues for individuals, making it a comprehensive tool for advancing personalized anti-cancer therapy. Personalized medicine in oncology is described as tailoring the most appropriate treatment for an individual person. The genomic-based drug response prediction promotes the personalized medicine in oncology. However, such genomic-based drug response prediction may not be so accurate in real clinic world for its lack of drug sensitivity confirmation in human biological samples. Given that GIC PDOs can be generated from individual patients with characteristic resemblance to the human original tumor tissues and enable the drug sensitivity testing in a meaningful time window, we believe that the combination of PDO technology with genomic profiling will be the more promising strategy for personalized therapy for GIC patients. Ongoing trials should be done to provide the solid validation for applying the drug testing of GIC PDOs to personalized medicine. Results of a first study comparing drug sensitivity results of GIC PDOs with the drug responses in the clinic are encouraging [53]. Optimizing the sensitivity, accuracy and robustness in drug sensitive testing platforms will successfully lead the GIC PDO-based personalized medicine to clinic. We also expect that technologies, such as microfluidics, may overcome the challenge of mimicking the TME in GIC PDOs, and finally help to achieve cancer personalized medicine.

In this review, we have summarized the establishment of biobanks of GIC PDOs and have highlighted the applications of GIC PDOs in personalized medicine. Challenges and opportunities are always coexisting. Despite current limits need to be addressed, the fast developed PDO model do have a positive influence for advances in Oncology. We expect that patients could really benefit from the PDO-based personalized medicine in oncology in the future.

## Abbreviations

5-FU: 5-Fluorouracil
ALI: Air-liquid interface
aGEA: Advanced gastro-oesophageal adenocarcinoma
ATT-I: Atractylenolide I
BC: Biliary cancer
BE: Barrett’ esophagus
CAC: Cholangiocarcinoma
CAFs: Cancer-associated fibroblasts
CAR: Chimeric antigen receptor
Ca-IIP: Intrinsically Immunogenic Properties
CBP: Carboplatin
CCK-8: Cell counting kit-8
CHC: Combined HCC/CAC
CLU: Clusterin
CML: Chronic myelogenous leukemia
CN: Copy number
CNV: Copy number variation
CPT: Camptothecin
CPT-11: Irinotecan
CPS1: Carbamoyl phosphate synthetase I
CRC: Colorectal cancer
CRC-SCs: CRC stem cells
CTG: CellTiter-Glo
DDP: Cisplatin
DDR: DNA damage response
DFS: Disease-free survival
DOC: Docetaxel
DPYD/DPD: Dihydrothymine dehydrogenase
EADC: Esophageal adenocarcinoma
EC: Esophageal cancer
EGD: Esophageal gastroduodenoscopy
EPI: Epirubicin
ESCC: Esophageal squamous cell carcinoma
Eto: Etomoxir
EUS-FNB: Ultrasound-guided fine-needle biopsy
FAO: Fatty acid β-oxidation
FC: Flow cytometry
FOLFIRINOX: Oxaliplatin, leucovorin, irinotecan, 5-fluorouracil
FOXO3-Cyt: FOXO3 cytoplasmic distributed
FSK: Forskolin
GBC: Gallbladder cancer
GC: Gastric cancer
GEM: Gemcitibine
GICs: Gastrointestinal cancers
HA-Coll sponge: Hydroxypropyl cellulose allyl conjugated with collagen
HCC: Hepatocellular carcinoma
HGF: Hepatocyte growth factor
HIPEC: Hyperthermic intraperitoneal chemotherapy
HLA: Human leukocyte antigen
HSP: Heat shock protein
ICI: Immune checkpoint inhibitor
ILK: Integrin-linked kinase
LGR5: Leucine-rich repeat-containing G protein-coupled receptor 5
LV: Leucovorin
MA: Malignant-ascites
MADOs: Malignant-ascites derived organoids
mCRC: Metastatic colorectal cancer
MDR: Multi-drug resistance
mGICs: Metastatic GICs
MMC: Mitomycin C
MS: Mass spectrometry
MTA-3: (4,5-dimethylthiazol-2-yl)-5-(3-carboxymethoxyphenyl)-2-(4-sulfophenyl)-2H-tetrazolium
NAC: N-acetylcysteine
NM: Not mentioned
No.: Number of samples
NVB: Vinorelbine
OA: Oncolytic adenovirus
OS: Overall survival
OXA: Oxaliplatin
PBC: Pancreato-biliary cancer
PBLs: Peripheral blood lymphocytes
PC: Pancreatic cancer
PCPS1: Carbamoyl phosphate synthetase I
PDAC: Pancreatic adenocarcinoma
PDO: Patient-derived organoid
PDOs-^Matrigel^: PDOs cultured in Matrigel
PDOs-^Sponge^: PDOs cultured in sponge
PDOXs: Patient-derived organoid xenografts
PDT: Photodynamic therapy
PDTXs: Patient-derived tumor xenografts
PGE2: Prostaglandin E2
PI: Propidium iodide
PLC: Primary liver cancer
pS6: Ribosomal protein S6
PSMD4: Proteasome 26S subunit non-ATPase 4
PTX: Paclitaxel
RC: Rectal cancer
Ref: Reference
TME: Tumor microenvironment
VLB: Vinblastine
VP: Etoposide
YAP1: The Hippo-Yes-associated protein 1
